# Coronavirus Disease 2019 Emergency and Cancer in the South of Italy: What's New for the Oncologist?

**DOI:** 10.3389/fmed.2020.00189

**Published:** 2020-05-06

**Authors:** Concetta Ingenito, Luciana Buonerba, Claudia Ferrara, Giuseppina Busto, Annamaria Libroia, Gianluca Ragone, Emilio Leo, Beatrice Savastano, Concetta Dello Ioio, Ferdinando De Falco, Simona Iaccarino, Luciano Tarantino, Mario Polverino, Giuseppe Di Lorenzo

**Affiliations:** ^1^Oncology Unit, Hospital “Andrea Tortora,” ASL Salerno, Pagani, Italy; ^2^Department of Medicine and Health Sciences “Vincenzo Tiberio”, University of Molise, Campobasso, Italy

**Keywords:** coronavirus disease, cancer, patients, treatment, management

## Introduction

After the outbreak of coronavirus disease 2019 (COVID-19) in Italy, 120,000 infected and 14,000 dead individuals were reported on April 3, 2020^1^. Following a decree of the president of the council of ministers on March 9, 2020, people who had moved to the north of the country for mainly work or study purposes returned to their families in the southern regions, thus bringing the infection to these areas. The occurrence of this event led to a need to develop strategies aimed at containing and recording the movements of patients entering hospital settings, and including cancer patients. Based on the scientific literature, 19.4% of coronavirus deaths have occurred in people with oncological comorbidities. This data indicates a connection between anticancer therapies and susceptibility to coronavirus, particularly among lung cancer patients. About 28% of patients are affected by this pathology (1, 2).

## Measures During The Pandemic and Results

Based on the abovementioned data, the Oncology Unit (OU) “Tortora” in Pagani, Italy has been implementing a series of measures to track cancer patients since the beginning of the pandemic. These measures are aimed at protecting healthcare personnel and patients themselves.

Adopted measures ensured the correct use of personal protective equipment (PPE) by all healthcare personnel and patients. In addition, to avoid overcrowding of corridors or rooms, patients were prevented from entering the ward with caregivers.

A very effective measure that is also being implemented is the use of a pre-triage, both by telephone and in person. Telephone pre-triages are performed the day before scheduled cancer therapy by contacting each patient and asking if they have had a fever or cough in the last 15 days, if they have been in contact with possibly infected people and by sending the blood analysis via e-mail.

Blood test results are a fundamental part of the telephone pre-triage as they allow us to view the patient's parameters in advance and decide whether or not to have them visit the hospital. In fact, based on blood test results, therapy is often postponed for up to 2 weeks to reduce the patient's risk exposure.

On the other hand, an in person pre-triage on the day of therapy provides an opportunity for further checks, including a second interview and temperature measurements. Moreover, according to the Campania Region official press release n°96 on March 30, 2020, which referred to decree n°45 of March 6, 2020, serological tests will be performed on patients during the in person pre-triage. Serological tests are quick qualitative tests that detect IgM or IgG anti-CoV antigens^2^.

For patients who require ordinary hospitalization, such as elderly patients, patients with higher comorbidity or testicular cancer patients who require several days of treatment, a preliminary evaluation of treatment changes, such as providing hormonal therapy instead of chemotherapy for prostate cancer patients, can be performed in a separate ward before hospitalization to avoid the infection patients or healthcare personnel (Table 1). Quick serological tests will also be performed on hospitalized patients to implement infection control during hospitalization days.

**Table 1:** Strategies for patient management.

**Pre-therapy strategies in Day- Hospital (out of hospital)**	**Pre-therapy strategies in Day-Hospital (in hospital)**	**Pre-hospitalization strategies (ordinary hospitalization)**
Evaluation of oncological- disease and treatment: If possible, a treatment delay of 1/2 weeks	Evaluation of oncological- disease and treatment: If possible, a treatment delay of 1/2 weeks	Evaluation of oncological disease and treatment: If possible, a treatment delay of 1/2 weeks Change to oral therapy (ex: prostate cancer)
Telephone pre-triage: Fever measurement Last 15 days of clinical history Sending of blood analysis by e-mail or fax	Pre-triage in presence: Fever measurement Quick serological tests for IgM or IgG anti-coronavirus' antigen research	Hospitalization of: Patients in therapy for several days (testis cancer) Elderly patients with comorbidity (for example colon, prostate/bladder cancer)
Online platform for patient registration to enable daily follow-ups by the doctors of general medicine	PPE	Evaluation of last 15 days of clinical history
		Pre-hospitalization ward
		Quick serological tests for IgM or IgG anti-corona virus' antigen research during hospitalization days
PPE		PPE

*PPE: personal protective equipment*.

Undergoing all health professionals and thus identifying any asymptomatic positives and then dividing the staff into weekly teams. At the end of each week, a swab is taken from each staff member and then tested to identify any infected members and another team will change them to ensure continuity in the service. In the OU in Pagani, for example, the medical team is composed of 8 doctors, 2 nurses, and 3 data managers, and thus, the team could be divided into two groups for each week (Table 2).

**Table 2:** Strategies for healthcare personnel.

**Organization strategies**
Basal ward for all healthcare personnel
Weekly team composed by:4 doctors 2 nurses 1 data manager
Monitoring patient's clinical values through the online platform
PPE

*PPE, personal protective equipment*.

Plans are also being made to create an online platform where all patients belonging to the OU can be registered. This platform could be accessed by each patient's doctor of general medicine (DGM) who could enter the patient's data and keep hospital records updated. In this way, the DGM could help manage the patient population, especially among risk groups such as cancer patients. DGMs could ring cancer patients daily and then update their online files, thus allowing oncologists to carry out remote consultations in case of minor illnesses that do not require a hospital visit. These calls could alleviate the suffering that can result from isolation and prevent unnecessary access to the hospital.

In the midst of a forced quarantine, and due to the therapies they are undergoing, people with the greatest psychological repercussions are likely patients who need treatment and attention the most.

By creating an online platform and call system, such patients would have frequent contact with their doctor, thus feeling comforted and followed-up with, even during such distressing times. All the above mentioned measures also help us control patient's movements in the hospital as well as manage their therapy in a way that reduces their risk exposure to the coronavirus. Indeed, telephone pre-triage creates a connection between patients and healthcare personnel that allows patients to communicate any feelings of anxiety or doubts.

However, the sending of blood analysis results is the most important tool for therapy management because it allows us to decide whether therapy is necessary or possible before patients visit the hospital. If it can be done without affecting oncological outcomes, therapy can be delayed by up to 2 weeks to avoid patient exposure.

Patients eligible for telemedicine are younger (69 vs. 73 years) but have a higher number of risk factors for a severe course of COVID-19 (3). Implementation of telemedicine will be critical for the management of follow-up visits and oral drug delivery, as currently done in several institutions nationwide (4).

Our experience using these methods is positive; in fact, we have reduced the number of patients accessing the OU only for advice or blood tests control. Thus, it is possible to apply better regional and national directives for the containment of coronavirus.

## Discussion

The Italian Medicines Agency (AIFA) has approved the protocol drawn up by Prof. Paolo Antonio Ascierto's team for the use of the monoclonal antibody tocilizumab, with the main objective of reducing the coronavirus death rate. Tocilizumab binds the IL-6 receptor, thus preventing it from binding and activation other cytokines that would damage the lung parenchyma^3^. In fact, SARS-CoV-2 binds to alveolar epithelial cells and activates the innate and adaptive immune systems, which results in the release of a large number of cytokines, including IL-6. In addition, because of the role of these pro-inflammatory factors, vascular permeability increases and a large number of fluid and blood cells enter the alveoli, resulting in dyspnea and even respiratory failure (5).

Remdesivir, an antiviral, is still not approved by AIFA, but it is administered for compassionate use^3^.

Both tocilizumab and remdesivir can provide some relief to COVID-19 patients; however, given that a vaccine is not yet available, the main strategy to fight the virus is prevention, especially for the most at-risk patients, such as cancer patients. Cancer therapies, such as chemotherapy and radiotherapy, suppress the immune system, thus exposing patients, especially those over 75 years of age, to a greater risk of infection. Most chemotherapies cause neutropenia, reduction in the number of neutrophils, which are the body's first line of defense (6).

In the context of the coronavirus pandemic, according to a recent report on the management of patients with renal, germ-cell, urothelial, and prostate cancer, chemotherapy should be delayed, while androgen receptor signaling inhibitors (ARSI) treatment should continue, thus acting as a chemotherapy replacement (7).

Further, based on a national survey, oncologists have determined that in the management of patients with genitourinary malignancies, and in an advanced disease setting, it would be useful to delay the initiation of treatment or consider interruption of second or further lines of treatment when associated with a lower clinical benefit (4).

In conclusion, the fight against coronavirus does not only depend on the individual's choices but on everyone's actions. We all need to work together to track patients, especially those who are asymptomatic, track their movements and study for therapy. The aim is to guarantee separate and safe pathways for patients with cancer (8).

In this context, the needs of cancer patients must be taken into particular consideration by studying strategies that allow following of their progress without putting them at risk. Alternatively, if patients are already positive, developing a protocol to fight the virus without reducing chemotherapy and/or radiotherapy, is necessary to avoid negative oncological outcomes.

## Author Contributions

CI, LB, and GD have written the manuscript. All authors have critically reviewed it.

## Conflict of Interest

The authors declare that the research was conducted in the absence of any commercial or financial relationships that could be construed as a potential conflict of interest.

## Footnotes

^1^http://www.protezionecivile.gov.it/ (accessed April 2, 2020).

^2^http://regione.campania.it/regione/it/news/primo-piano/covid-19-da-domani-test-rapidi-per-gli-operatori-della-sanit (accessed March 30, 2020).

^3^Official site of AIFA, section dedicated to experimentation for the treatment of SARS-CoV-2 https://www.aifa.gov.it/sperimentazioni-cliniche-covid-19.
